# Exploring nurses' experiences of social media and in-person educational interventions for professional development: a qualitative study

**DOI:** 10.1186/s12912-022-00903-4

**Published:** 2022-05-24

**Authors:** Seyedeh-Somayeh Kazemi, Sedigheh-Sadat Tavafian, Alireza Hidarnia, Ali Montazeri

**Affiliations:** 1 Department of Health Education & Health Promotion, Faculty of Medical Sciences, Tarbiat Modares University, Tehran, Iran; 2 Health Metrics Research Center, Iranian Institute for Health Sciences Research, ACECR, Tehran, Iran; 3 Faculty of Humanity Sciences, University of Sciences & Culture, ACECR, Tehran, Iran

**Keywords:** Content analysis, Qualitative study, Education, In-person, Social media, Nurse

## Abstract

**Background:**

Nurses play an important role in health promotion, prevention strategies, and care. Therefore, nurses need to obtain and update their knowledge and skills via appropriate strategies. This study aimed to explore nurses’ experiences of receiving social media and in-person education to integrate the findings into practice.

**Methods:**

This was a qualitative study using the directed content analysis approach. A sample of nurses with previous experiences of receiving social media and in-person education participated in the study. They were asked to express their experiences and indicate their preferences. The data were collected based on individual semi-structured interviews.

**Results:**

In total 15 participants took part in the study with a mean age of 40.6 ± 8.93 years and work experiences of 15.3 ± 9.21 years. During the process of content analysis, three main themes emerged: Approaches to nursing education and its adoption in the health system, Achieving effectiveness and efficiency in nursing education, and Health care policy and facilitating pathways for nursing education. Participants indicated several barriers to attending an educational program, including motivation, workload, time and place, and hospital politics.

**Conclusion:**

Overall the findings suggest that regardless of any methods of education nurses cannot actively engage in the educational interventions while on duty. However, the findings suggest that nurses believe that the social media approach might be superior in reducing barriers and making the educational interventions work better.

**Supplementary Information:**

The online version contains supplementary material available at 10.1186/s12912-022-00903-4.

## Background

As the most important part of the health system employing a comprehensive health approach, nurses significantly affect the effectiveness of the healthcare system and play an important role in the promotion of health, prevention of diseases, and treatment and care [1]. This issue is in favor of individuals, societies, and populations in the healthcare center. The importance of the role of nurses in investments in the healthcare area and the improvement of economic growth of communities is undeniable [2]. Thus, focusing on developing the professional knowledge and skills of the nursing workforce is very important.

Professional development that involves training during working hours or beyond is an important issue for nursing personnel [3]. Approximately 90% of the universal health care is provided by nurses [4, 5], so the professional development of nurses can be useful in patient care, the efficiency of organizations, and the promotion of nurses [6]. As such implementing educational programs for nursing personnel during working hours or out-of-hours are essential. Different strategies have been employed to implement professional development through educational programs using electronic (mobile, message, website), and in-person (lectures, role-playing) education methods.

An education method comprises the principles and methods used to enable learning in the learner. For a particular education method to be appropriate and efficient, it has to be relevant to the characteristics of the learner and the type of learning it is supposed to bring about [7]. Researchers of face-to-face or in-person education argued that effective action requires more than learning new tools and techniques, and must also include exploration of the theories and values which underpin facilitation practice [8]. The focus should also be on the personal qualities and attendance that facilitators bring to the group [8, 9]. Some studies have shown that educational programs based on social media might be more helpful compared to other forms of education [10, 11].

Rapid and innovative advances in participative Internet communications referred to as “social media,” offer opportunities for modifying health behavior. People of all demographics are adopting these technologies whether on their computers or through mobile devices and they are increasingly using these social media for health-related issues. Although social media have considerable potential as tools for health promotion and education, these media, like traditional health promotion media, require careful application and may not always achieve their desired outcomes [12]. Some studies show social media intervention has been successful in the management of low back pain [13–15], Type 2 Diabetes [16], self-efficacy [17]. Social media can support nurses in numerous ways on a personal level. Moorehead et al. highlighted several such as, increased number of interactions with others, shared and custom health content, access and availability of health information, and peer and social support [18].

Social media includes different online tools and some have been used in health promotion, such as Web Logs, Websites, Message boards, Short message services, Social networking sites, etc. [12]. As technology is rapidly changing, so is pedagogy within nursing education. In recent years, using the flexibility of the Internet provides a good opportunity to extend scientific content and enhance learning in general [19]. A qualitative study on the consequences and factors affecting work-related low back pain in nurses found that nurses could not attend the educational program continuously based on a variety of causes, such as a heavy workload, lack of time, or lack of staff [20]. It seems nursing personnel is among those who could benefit from social media including website, app given that they have growing educational needs and skills and at the same time they are dealing with overloading tasks scheduling [21].

The PRECEDE–PROCEED model is often used in health education and health promotion. This model is a cost–benefit evaluation framework proposed in 1974 by Lawrence W. Green that can help health program planners, policymakers, and other evaluators, analyze situations and design health programs efficiently [22]. Based on the study by Green and Kreuter, analyzing situations and designing and implementing educational programs efficiently can be classified in enabling factors and administrative and policy diagnoses. Enabling factors are those characteristics of the environment that facilitate action and any skill or resource required to attain specific behavior [23]. They include programs, services, availability and accessibility of resources, or new skills required to enable behavior change. The administrative and policy diagnosis phase focuses on the administrative and organizational concerns that must be addressed before program implementation. This includes assessment of resources, development, and allocation of budgets, looking at organizational barriers, and coordination of the program with other departments, including external organizations and the community [24].

In this study, we intend to explain the advantages and disadvantages of the two educational approaches. In other words, we construed and explained the presentation of educational content or intervention via two approaches of in-person and social media from the perspective of nurses based on PRECEDE model. As well, we construed the governing policies in our country hospital in facilitating the provision of education through two approaches.

Because education plays an important role in nurses’ career advancement, the present study aimed to identify and explore nurses’ experiences of two approaches of education (social media such as website, App, and in-person education) using a qualitative method.

The study sought to answer the following questions:

Which educational approach do nurses prefer? Why?

Which educational approach is more appropriate and effective?

Which approach is in line with hospital policies?

## Methods

### Study design

As part of a trial [25], a qualitative study was conducted to explore nurses' experiences of social media and in-person education approaches. The study used the directed content analysis approach. The goal of a directed content analysis approach is to validate or extend conceptually a theoretical framework or theory [26]. Existing theory or research can help focus the research question and it can help researchers begin by identifying key concepts or variables as initial coding categories. This approach was employed by Hsieh and Shannon in 2005 [27]. Content analysis using the directed approach employs a more structured process than in a conventional approach [28]. As such it is appropriate to use existing theories or prior investigations about the phenomenon under study.

### Participants

Participants included nurses working at relevant hospitals. Inclusion criteria were: at least one year of work experience in nursing, previous experience of receiving social media, and in-person education. The study was approved by Tarbiat Modares University of Ethics Committee for Health Research Ethics (IR. TUM. REC. 2017/545), and all participants provided written consent. As well as, the researcher reminded commitment to ethics in research, such as secrecy, preservation of anonymity, permission to leave the study, and interruption of the interview if desired.

### Data collection

The data were collected based on individual semi-structured interviews [29, 30]. The Interviewees in this study were nursing personnel. The recruitment of participants for the interviews was based on purposive sampling. Decisions regarding the number of individual interviews were determined by data saturation [31]. The original interviews were conducted by the main researcher (SSK) between August 2017 and March 2018 and lasted between 35 to 50 min. All the interviews were conducted in a private room. In the first, the researcher explained the study goals and reasons for doing the research to the participants. To ensure consistency, each interview followed the same semi-structured format, using an interview schedule consisting of open questions, all interviews were recorded and transcribed. The interview guide consisted of open-ended questions based on subcategories of the enabling, administrative, and policy factors of PRECEDE model to allow respondents to fully explain their own experiences. The questions such as "What do you think about social media and in-person education approaches?/ Which approach is in line with hospital policies?" Likewise, they were asked to express their experiences and indicate their preferences.

### Data analysis

The study used the directed content analysis approach. The data analysis process was carried out simultaneously by collecting data [32]. Semi-structured interviews were tape-recorded, transcribed verbatim, the transcript read several times, and parts of the text highlighted, that were relevant to the study goals. The highlighted passages were broken up into semantic units. Then investigators identified meaning-units from each transcript and performed initial coding according to the participants' phrases. Any text that could not be categorized with the initial coding scheme was given a new code. Initial codes were identified by at least two co-investigators, and codes were then categorized according to similarities and differences [31]. An initial analysis created 20 excerpts (sections of text). The data were independently coded by another researcher with in-depth knowledge to explore consistency. The second researcher also created 22 extracts. Transcripts, codes, and categories were read several times by various members of the research group to exhaust identifiable major themes. During this process, data were reduced from text to codes and categories. Based on the type and breadth of a category, researchers identified subcategories with subsequent analysis. When the coding assignments were done for both researchers, have reviewed the three assigned codes from expected outcomes, such as presentation and adoption of education, effectiveness, and efficiency of education, policymaking, and facilitators for education. All coding steps for interview passages were also managed using the MAXQDA 10 editing software.

### Validation

The evaluation criteria for establishing the trustworthiness of qualitative data were also considered [28]. There was constant comparison within and across categories and across interviews where each code or category was checked against the rest of the data to establish and refine categories that reflected the nuances of the data [33].

Credibility is the equivalent of internal validity in quantitative research and is concerned with the aspect of truth-value. One of the strategies to ensure credibility is member checking [34, 35]. All transcripts of the interviews were sent to the participants for feedback. Further, halfway through the study period, a meeting was held with those who had participated in the interviews, they corrected the ‘wrong’ interpretation.

Dependability, related to reliability in quantitative terms, occurs when another researcher can follow the decision trail used by the researcher [36]. The text of the interviews, the relevant codes, and the emerged themes were sent to two researchers outside the study who were familiar with qualitative research. Outside researchers examined the research process and the data analysis to ensure the findings were consistent and could be repetitive. Then their complimentary comments were used in the analysis of the data. Further, at a meeting, we discussed the interpretation and presentation of the research findings of uncomplimentary until achieved a common result. As well, we used peers to participate in the analysis process to enhance the original findings.

Confirmability that the findings were based on participants’ responses and there were not any potential bias or personal motivations of the researcher [34]. We have tried to use the participants’ narratives and words rather than potential researcher biases. To establish confirmability, the researcher provided an audit trail, which highlighted every step of data analysis that was made to provide a rationale for the decisions made. This helped establish that the research study’s findings accurately portrayed participants’ responses.

## Results

### Characteristics of the participants

The original data collection comprised a series of interviews with a sample of 15 project participants. Most were female (*n* = 11) and their age ranged from 27 to 52 years old (40.6 ± 8.9). They have been employed as a nurse for at least one year (15.33 ± 9.2 years). Participants included nurses working in hospitals affiliated with Mazandaran University of Medical Sciences. The characteristics of participants are presented in Table 1.

**Table 1:** Demographic characteristics of the participants in In-depth interviews

Individual Characteristics	Total (15) n (%) or mean ± SD
**Age, years**	40.6 ± 8.9
**Gender**	
Female	11 (73.3)
Male	4 (26.7)
**Profession**	
Head Nurse	3 (20)
Nurse	12 (80)
**Years in Profession**	15.33 ± 9.2

### Themes, categories, sub-categories

The study obtained 3 themes, 9 categories, and 22 sub-categories (Tables 2, 3).

**Table 2:** The process of formation codes and sub-themes

Semantic unit	Codes	Sub-themes
According to working conditions and crowded units using the app is much better. Whenever I want to use it at work or out of work, at home and anywhere. Instead of reading books, we use more mobile phones. Because everywhere is with us. Maybe I cannot attend classes because of leave or work shift, but I can use the app at any time. Because it's available	Using the app at rest time Using the app at home and at work.	Access to education without time limitation Access to education without space limitation
The staff is low and the workload is high. Sometimes, for example, maybe there are 8 patients, with 4 nurses and patients put intubation. Well, we cannot be in the class with so much sick and low personnel and staff	Supporting or managing a large number of patients in the units	The high workload in nursing Work pressure
When one thing or something like exercise forgot, we can go back to the app and use it, but after the in-person education, we do not have any document or paper that in the case of forgetfulness, we can refer to it	Forget information because it does not repeat	Remember and repeat the content

**Table 3:** The process of formation sub-themes, themes, and main themes

Sub-theme	Theme	Main theme
- series education by the head nurse -Provide conference by units’ nurses -Implementation of education through social media	Performing the program by the staff	Approaches to nursing education and its adoption in the health system
-Practical education -Skill assessment during education	Skill Learning	Achieving effectiveness and efficiency in nursing education
-Ask question -Participation of participants	Interactivity in education	
-Remember and repeat the content -Use content over time	Remembrance for restoring of information	
-The material and spiritual motivation of participating in education -Managers' support of education -Consider reward and punishment	The importance of motivation in education	Health care policy and facilitating pathways for nursing education
-Lack of access to education without time limitation -Lack of access to education without space limitation -Lack of access to a lot of educational content -Lack of share information	The time limit for easy educational access	
-The high workload in nursing -Work pressure	Lack of staff	
-Non-payment of education -Low cost of education through social media	Lack of funding for education	
-Holding in-person education on limited dates -Space restrictions for in-person education	Program Failure over time	

Approaches to nursing education and its adoption in the health system.

Nurses acknowledged that most of the time, in-person education is not provided by an education specialist. So, this theme was derived from performing the program by the staff working in the hospitals, such as the educational supervisor, head nurse, or different sectors' nurses. In this case participant 4 (head nurse, age range 50–60) said:

“*Some of the time, education is provided by the staff or nurses. The supervisor teaches head nurses and the head nurse educates sector nurses in the form of a category education to the category. Since different departments need to have internal education, some education will be provided by nurses of the same department as a conference. But due to the bustling ward and a large number of patients, not all colleagues can attend the training at the same time. If education is implemented through the app or social media, then there is no need to provide education by nurses or other staff.*”

Achieving effectiveness and efficiency in nursing education**:** refers to whether or not a specific set of resources has a positive effect on achievement and, if so, how large this effect is. This main theme was extracted from three sub-categories as follows:

- Skill Learning: The effect of education on increasing knowledge, practice, and skill was identified, and the role of practical education in promoting behavior skills was emphasized. In this regard, participant 3 (nurse, age range 20–30) said:

*“The system thinks in-person education is better than social media. Because in-person education can provide practical examples, nurses can ask questions and issues related to the subject of education. Also, pre-test and post-tests are taken in an in-person education, and the nurse's performance is evaluated. While the system can be designed for education via social media in such a way that contains educational videos practical examples in the form of videos, photos, animations, etc. And the educational supervisor monitor how many percentages of the staff used the program and training. As well, evaluate our knowledge and performance through the website."*

- Interactivity in education: Interactivity in learning is viewed as a socio-constructivist process and can be communicated among content and participants [37]. Most participants had referred to interactivity in In-person education because In-person education can prepare an adequate situation to ask questions and stimulate communication between participants. Since nurses have received educational content via CD or pdf files sometimes, they thought education via social media is one-way and interactive communication is not possible. So participants emphasized two-way communication in education and its interactive social media. Participant 15 (nurse, age range 40–50) said:

*“When staff gets together, it is very good. Because they can be exchanging ideas. Also, there are a lot of questions for each of us, and maybe at that moment don't work the memory to ask. Persons can be actively participation in education and do some practical work. But I think in interactive social media, we can talk and express our opinions."*

- Remembrance for restoring of information:

One of the nurses' concerns was the oblivion of the content learned over time. Most participants believed that an appropriate education program should be repeated over time, otherwise it will be forgotten. In this regard, participant 1 (nurse, age range 50–60) said:

*"One of the disadvantages of in-person education is receiving little information and content and it only is at one time and the information learned does not repeat over time. Thus, the content learned will be forgotten. But I think one of the benefits of education through the app or social media is receiving a lot of information and content at different times, and also reminding and repeating the content. If we forget about it over time, we can refer to the app."*

Health care policy and facilitating pathways for nursing education.

This theme was extracted from five sub-categories as follows:

- The importance of motivation in education:

Considering that educational programs are part of hospital accreditation measures, lack of motivation, and disinterest by nurses, also lack of support and reward by managers prevent nurses from participating in face-to-face education. Thus, these factors lead to not attending the class on time, leaving the class out earlier, lack of taking notes, and not paying attention to education. Participant 5 (nurse, age range 30–40) on the role of material and spiritual motivation said:

*"If the education program is out of work hours, nurses don't like in-person education and prefer to receive training virtually. Because the time of education will not be considered as a part of our overtime hours. The encouragement certificate for work promotion was not considered. While the certificate can have a positive impact on the annual performance appraisal."*

- The time limit for easy educational access:

Getting easier to access education and having no time limits are basic nurses’ rights. But based on the analysis obtaining in-person education and its access was less possible for nurses. In this regard, participant 9 (nurse, age range 20–30) said:

*“When only one date is set for an educational program, maybe we can't attend. For example, perhaps there were many patients at the same time and there aren't many nurses in the sector. Certainly, we cannot participate in education. It is better to set a few dates for an education topic so that nurses can participate in different dates or send via social media. In this way, we can read the contents when the unit is not crowded or at rest time. "*

Participant 11 (nurse, age range 30–40) said:

*"I think the advantage of social media is most people can access this type of education and share information. We can use this program whenever we want, and we don't have to go to the hospital from home. There is no time and space limit. Even at rest, at work and home, at any time of day, we can use this type of education. We can also have access to a lot of educational content."*

- Lack of staff: Most participants referred to the lack of staff in different sectors. They said these factors lead to working pressure. Working pressure and high workloads are seen as the main barriers to attending in-person education programs. Therefore, nurses didn't have enough time to attend in-person education and expressed their tendency to use educational programs based on social media. In this case, participant 2 (nurse, age range 30–40) said:

*"We are faced with a lack of staff. So that one person should work instead of two or three persons. Thus, the load of our work is very high, and we have to tolerate a lot of pressure."*

- Lack of funding for education:

Data analysis found one of the barriers to in-person education is the cost of education implementation. Thus, one of the most important concerns of education executives is the financing of education costs (such as the payment of the educator, catering fees, educational equipment, and adequate space for education). Participant 6 (nurse, age range 30–40) said:

*"One of our educational problems is a lack of funds. The hospital's operating personnel says you should find a sponsor for the educational program. In-person education is economically not desirable because of the commute and payment to the educator. But education through social media is just an app that's installed on the mobile phone and does not require commuting, educator fees, or catering, even it has not needed a place for education."*

- Program Failure over time:

The data showed some factors that lead to program failures over time. Such as lack of suitable education space, lack of proper education, no having trainer or educator. In this regard, participant 13 (nurse, age range 30–40) said:

*"Perhaps faculty members educated their students in the hospital and not at college or maybe there was a retraining class. So the educational supervisor will coordinate with the unit of education a few days before the education. But, sometimes the education program maybe to be canceled due to a lack of appropriate location or space."*

## Discussion

Regarding the decisive role of the educational method in nursing education, this study aimed to explore nurses' experiences of the education methods context based on the in-person and social media methods through a qualitative study. The findings from this survey indicated due to the nature of the nurses' work, the educational methods and easy access to education can be a considerable role in nurses' education, particularly in promoting professional and occupational skills.

One of the main themes extracted was "Approaches to nursing education and its adoption in the health system" i.e. the educational program performed by the hospital staff. This theme illustrated the provision of the educational program by the staff, as there was no specialist educator personnel to run the program. This was due to a lack of financial support for a specialist educator. The effectiveness and success of the education process depend on the educator's professional skills. Occasionally, education is provided by an educational supervisor, or nurses such as series education, which is only as a task, although some education may be successful. Geravandi et.al study finding showed that education was effective by the clinical supervisor [38].

Nursing education plays a major role in fostering the mind to think in clinical practices, different situations, and the establishment of communication between events, decision making, and conceptualization to promoting community health [39]. Nurses reported training as an effective factor in their ability to gain knowledge, skills, and self-efficacy in different areas. For instance, a study showed the effect of education to promote nurses' ability on patient care improvement and increase the quality of health care [10].

Nurses emphasized interactive education. Nurses in in-person education can ask questions, receive answers, and there is a relationship between the educator and learner. Nurses showed their tendency to use interactive social media. That means they could write their question if they could not understand the content or can comment on website/app content. The use of online courses also provides a good opportunity for expanding scientific content and increasing the depth of learning because of the flexibility provided. A study concluded that e-learning can be considered as a supportive and complementary method in nursing education [40]. Indeed, using social media for sharing information and connecting with their colleague, also for peer support, peer learning, and participation engagement seem effective and beneficial.

Participants also had comments on barriers and facilitators of education. Nurses acknowledged that they could not access the content if they did not attend the training. They believed they could access information and educational content using a social media method at any time, anywhere. This supports a study by Thiele which showed access to more information, taking responsibility for learning by learners, and access to educational content at any time by nursing students were achieved [41]. This finding is also consistent with Buckley et al. results who theorized that the facility of access to educational content in the Web-based method leads to increased satisfaction of learners [42]. Nurses can use a variety of social media programs to advance their personal and professional goals. Nurses can access information for their workplace or personal lives, connect with colleagues, share information about best practices, and advance health through personal and professional means [43, 44].

Another side, the data depicted a lack of motivation as one of the barriers to training participation and can lead to not paying attention to educational content, and also leaving the class before the education finished. In similar studies, inadequate motivation for education, lack of support, and encouragement for nurses to participate in education are mentioned [45, 46]. Thus, based on policymaking, managers are recommended to motivate nurses to attend in education, such as financial support, reward.

High workload, shortage of personnel, and lack of proportionality of the number of patients to the nurse in different units impede attending in-person education. Other studies also pointed to the high amount of work and work pressure on colleagues for attending continuing education as a barrier [40, 47]. As well, the data indicated inadequate financial support of the organization or lack of funding for education to create a suitable platform for education, in particular, in-person education. So that hospitals should run educational programs following the Ministry of Health instructions. However, the cost of education and the necessary preconditions for the implementation of education are not provided. This issue has always been one of the most important problems for education executives. In similar studies, inadequate funding by the organization was a major barrier to nurses' attendance in professional education [45, 48]. While nurses believed that directors' support from nursing staff would have a key role in their productivity. Furthermore, nurses represent education social media is appropriate because of increased quality of learning, ease of access to a high level of information at a limited time, reduced costs and also, family conditions, fatigue due to long work hours, and a lack of adequate human resources.

### Limitations, implications, and perspectives

The main limitation of our study is related to data collection. Some nurses were reluctant to participate in the study because of their high workload and lack of time. To overcome this limitation, the researcher asked nurses to choose the time of the interview according to their working conditions. Other limitations of this qualitative research include the following:

The interview process was time-consuming.

It was not possible to reach all the participants.

The interview required the consent and willingness of the respondent, the patience and perseverance of the interviewer.

Sometimes, participants were reluctant to express explicit opinions.

### Strengths

Despite its limitations, our study has had several characteristics of value. Firstly, our results provided a platform from the nurses' standpoint to evaluate educational approaches. As well as, it is recommended policymakers assess resources, development, and allocation of budgets, organizational barriers, and coordination of the program according to target groups.

## Conclusion

This study provided some advantages and barriers of the two educational methods from the nurses’ perspective. The choice of educational methods in nursing education is very important. Currently, despite inadequate motivation, insufficient support, and much workload, nurses are not motivated enough to attend in-person education. Therefore, it is necessary to establish a suitable framework for the implementation of the education process for nursing personnel. Indeed, it seems that applying educational methods based on interactive social media might be helpful.

## Supplementary Information

**Additional file 1.**

## Data Availability

The data will be available from the corresponding author on request.
