# Supplementary material for: Exploring nurses' experiences of social media and in-person educational interventions for professional development: a qualitative study
Source: BMC Nurs. 2022 May 24;21:126. doi: 10.1186/s12912-022-00903-4 (PMC9128214; doi:10.1186/s12912-022-00903-4)
Supplement: Supplementary file 1 — Additional file 1. [file 12912_2022_903_MOESM1_ESM.docx]

**Additional file 1. The questionnaire in English**

**Development of the questionnaire**

1- How do you plan to run a workshop in your hospital?

2- What is it like to hold education workshops in terms of time and place?

3- Are the educational programs run by the staff of the department or educator?

4- Does the hospital financially support the educational programs?

5- What do you think are the restrictions in holding the educational programs?

6- Can the executive, managerial and policy-making factors of the hospital prevent the holding of the educational programs? Especially for nurses whose work shifts are changing.

7. How do policy-making factors facilitate the implementation of educational programs?

8- If you need to hold educational programs serially, for example, once a month or once a season in the hospital, what do you think we should do?

9- Do you need to coordinate with the hospital management, unit' metron, or head nurse?
